# Robust and Accurate Anomaly Detection in ECG Artifacts Using Time Series Motif Discovery

**DOI:** 10.1155/2015/453214

**Published:** 2015-01-22

**Authors:** Haemwaan Sivaraks, Chotirat Ann Ratanamahatana

**Affiliations:** Department of Computer Engineering, Chulalongkorn University, Phayathai Road, Pathumwan, Bangkok 10330, Thailand

## Abstract

Electrocardiogram (ECG) anomaly detection is an important technique for detecting dissimilar heartbeats which helps identify abnormal ECGs before the diagnosis process. Currently available ECG anomaly detection methods, ranging from academic research to commercial ECG machines, still suffer from a high false alarm rate because these methods are not able to differentiate ECG artifacts from real ECG signal, especially, in ECG artifacts that are similar to ECG signals in terms of shape and/or frequency. The problem leads to high vigilance for physicians and misinterpretation risk for nonspecialists. Therefore, this work proposes a novel anomaly detection technique that is highly robust and accurate in the presence of ECG artifacts which can effectively reduce the false alarm rate. Expert knowledge from cardiologists and motif discovery technique is utilized in our design. In addition, every step of the algorithm conforms to the interpretation of cardiologists. Our method can be utilized to both single-lead ECGs and multilead ECGs. Our experiment results on real ECG datasets are interpreted and evaluated by cardiologists. Our proposed algorithm can mostly achieve 100% of accuracy on detection (AoD), sensitivity, specificity, and positive predictive value with 0% false alarm rate. The results demonstrate that our proposed method is highly accurate and robust to artifacts, compared with competitive anomaly detection methods.

## 1. Introduction

Electrocardiogram (ECG or EKG) signal is a time series data sequence which represents electrical impulses from myocardium. An ECG signal is recorded from many electrodes which are attached over skin. Most physicians prefer a use of ECG as a noninvasive tool to detect and diagnose cardiac diseases. The two important characteristics of an ECG signal are its multiple signal recordings from various positions of myocardium and its periodic waveform [1] synchronized with a cardiac cycle. A normal ECG consists of five morphology segments, that is, PQRST waveforms that correspond to electrical conductivity through the whole cardiac cycle. One cycle is composed of depolarization and repolarization from an atrium to a ventricle. ECGs from different leads may be morphologically different depending on the vector of the heart. The ECG morphology in each lead reflects the electrical activity in each segment of the heart. Therefore, the multilead ECG could be used to interpret the electrical activity of the whole heart and is very useful for abnormal myocardium detection (for more details, see [2–4]).

**Figure 1:**
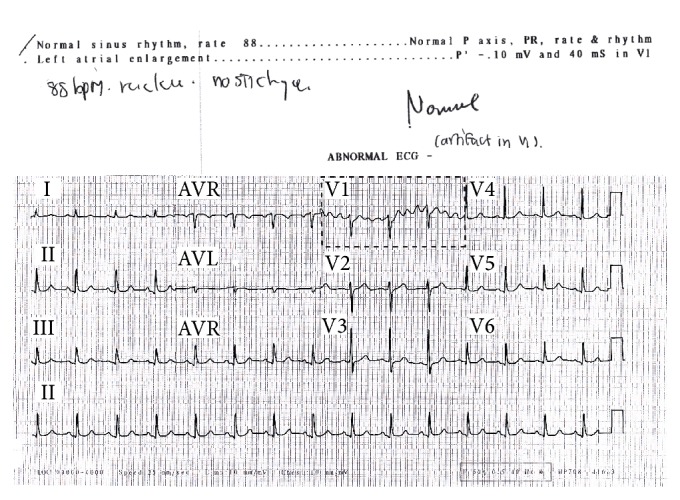
Sample of a 12-lead ECG recorded from a normal patient. ECG artifacts occurred in lead V1. Therefore, an ECG machine interprets these ECGs as abnormal ECGs whereas cardiologist diagnoses them as normal ECGs with artifacts (see the handwritten note).

ECG anomaly detection therefore has increasingly become a popular task among researchers and practitioners [5–13]. It has been used to detect any time periods of unusual ECG beats. The accuracy of the anomaly detection method directly reflects the result of the cardiac disease detection and diagnosis. However, existing algorithms that have claimed to achieve high accuracies still suffer from false alarm results [13, 14]. False alarm results typically occur because the algorithm detects some ECG artifacts as anomaly beats; in fact, some ECG artifacts are just normal beats. ECG artifacts result not only from the electrical activity of the heart alone but also from noise interference as illustrated in lead V1 in Figure 1.

Although artifacts are common in typical recordings, they pose serious problems in medical treatment as referred in several medical research works due to the impossibility of eliminating artifacts [14–16]. A number of works in medical domain have raised this concern to physicians to be aware of the artifact problem.

To study the problem of ECG artifact and false alarm result, we give questionnaires to physicians who have worked closely with ECG machines. We discovered that ECG machines often misinterpret ECG artifacts as anomaly, triggering excessive alarms to both patients and physicians on bedside monitors. Nonexperienced physicians then need to consult with cardiologists to manually reanalyze the results by considering all 12-lead ECG signals. In addition, clinical correlation is demanded and re-recording of a patient's ECG may be needed. Therefore, false alarm results not only waste valuable time for cardiologists but also lead to misdiagnosis for nonexperienced physicians.

**Figure 2:**
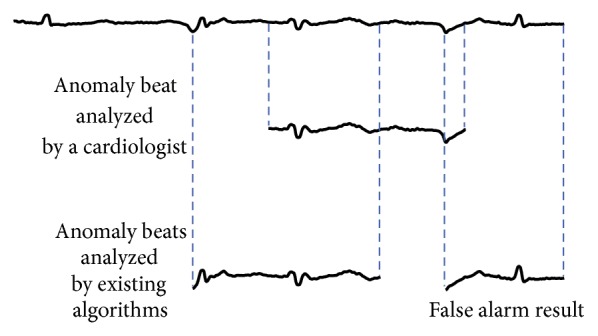
Sample of an ECG with an anomaly beat (top). An analysis by a cardiologist indicates one single anomaly beat (middle), whereas an existing algorithm indicates two anomaly beats, one of which is a false alarm (bottom).

Existing anomaly detection algorithms [5, 17–21] still suffer from false alarm detection results, as illustrated in Figure 2.

To reduce the false alarm results caused by noise interference, various noise reduction methods have been applied to preprocess the ECG signals [22–24], such as independent component analysis [25], wavelet transform [26–28], morphological filter [29], empirical mode decomposition [30, 31], adaptive filter, and artificial neural networks [32].

These techniques are highly effective in removing artifacts frequencies or components that are very different from those of ECG morphology (see Section 3.2). However, they still cannot handle ECG artifacts that have appearances similar to ECG morphology. These techniques could distort the ECG waveform and easily lead to misinterpretation [33, 34] because of the failure to distinguish between ECG artifacts and real ECG morphology.

In this paper, we propose a robust and accurate anomaly detection algorithm (RAAD) for ECG to reduce the false alarm rate. The challenges of this work include how to distinguish ECG artifacts from real ECG morphology in the case (1) where ECG artifacts could have numerous and uncertain shapes and (2) where the shapes of the waveform in each individual lead in each patient are all different.

To deal with the variation of ECG, our algorithm utilizes time series motif discovery to first determine frequent patterns in the ECG. In addition, we use expert knowledge to design every step of the algorithm to guarantee the conformity with the interpretation of cardiologists.

To evaluate the algorithm, brute force discord discovery [21], HOT SAX algorithm [35], and BitClusterDiscord [36] are compared with our work on several datasets from Physionet [37]. Our experiments are twofold: (1) to compare the detected anomaly subsequences with real anomaly beat diagnosed by cardiologists and (2) to evaluate algorithms using five statistical measurements which are accuracy on detection (AoD), sensitivity, specificity, positive predictive value, and false alarm rate.

## 2. Related Works

### 2.1. Definitions

To better understand terminology in this paper, the following definitions are provided.

**Definition 1 .**

An ECG in one single lead is a* time series T*, where a time series *T* of length *n* is an ordered set of real number sequence *t*_1_, *t*_2_,…, *t*_*n*_.

**Definition 2 .**

An ECG* subsequence C*_*i*_ of length *m* is a subset of an ECG with the starting position *i* consecutively *t*_*i*_, *t*_*i*+1_, …, *t*_*i*+*m*−1_, where 1 ≤ *i* ≤ *n* − *m* + 1.

Other terminologies in motif discovery process follow those in [38].

### 2.2. Related Works

Several research works have utilized time series mining technique in anomaly detection. Anomaly detection algorithms generally define an anomaly or a discord as the most unusual subsequences in a long time series. Many works utilize discretization methods to avoid noise inferences and use distance measures with pruning methods to measure the dissimilarity among subsequences. For instance, HOT SAX algorithm [21] bases itself on the representation of symbolic aggregate approximation (SAX). The algorithm has shown a great potential for extending and applying to many other works [39, 40].

Most recently, Sanchez and Bustos [41] proposed a new algorithm for efficient discord discovery in time series data based on HOT SAX algorithm. The aim of the algorithm was to reduce the time complexity of the algorithm. However, an ECG dataset was used in the experiment without showing any anomaly result or the parameter settings. Another work [20] aimed to improve an effectiveness of the anomaly detection algorithm in time series data but only focused on clean signals and did not concern much about the issue of noise within the signal. Some anomaly detection algorithms specifically for ECG data [5, 42] have been proposed based on machine learning technique, so training process is needed. However, ECG artifacts are known to be unstructured; in other words, they have numerous and uncertain shapes and the shapes of the waveform in each individual lead in each patient can totally be different. Therefore, it is difficult and impractical to collect a large number of training data with ECG artifacts. Another recently proposed work,* BitClusterDiscord* algorithm [36], bases itself on bit representation clustering, aiming to improve the effectiveness of the algorithm without requiring a training model. However, these algorithms do require the length of an anomaly beat as an input from users, which could be impractical for real ECG anomaly analysis.

To deal with the problem of predefined input length, many works have been proposed. For example, in [38, 43], minimum description length or MDL is used to automatically discover intrinsic features and is utilized to detect anomalous ECGs. An adaptive window-based discord discovery (AWDD) [19] has been proposed to detect ECG anomaly. It has been developed from brute force discord discovery (BFDD) [35]. This work uses R point in every 40 seconds to extract a variable-length ECG.

Apart from the abovementioned works, there are numerous research works relating to anomaly detection in both single-lead ECG and multilead ECG. However, these works are not practical due to the following reasons.

**Figure 3:**
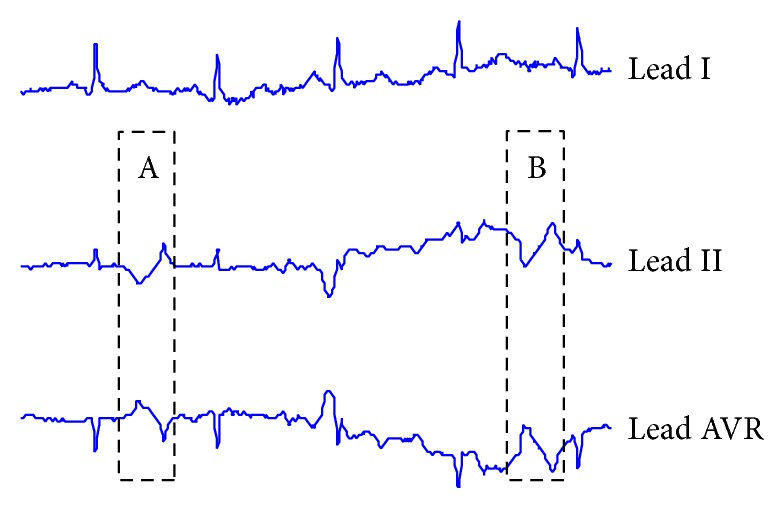
Sample of 3-lead ECG signals from a normal patient, which interfered by artifacts that make them appear to be anomalous, as shown in areas A and B.

1. Many works [5, 8, 18, 20, 39–42, 44–47] use only clean ECGs or ECGs with simple artifacts. In fact, real ECG data are always contaminated by noise and contain various types of ECG artifacts. Therefore, many false alarm results often occur when these works are applied to real ECG data, as shown in Figure 3.
2. Many works [21, 35, 36, 48] required a fixed length of result as an input parameter from users. To determine the length, it is, in fact, very difficult to know what the proper length is. Although some works [19, 20, 38, 43, 47, 49] have presented their algorithms with variable lengths of results, and the length of the results may not be consistent with the length of actual cardiac cycle. Therefore, the result may not properly cover the cardiac cycle or morphology which is crucial for diagnosis.

The abovementioned problems are very challenging. To the best of our knowledge, our work is the first anomaly detection technique that works for ECG artifacts with the main focus on reducing false alarm rates, whilst retaining high accuracy.

## 3. Background

### 3.1. 12-Lead Electrocardiogram

Electrocardiogram [2, 3, 50] is a graphical signal that represents the electrical activity generated by the cardiac muscle. We usually plot an ECG signal as an amplitude or voltage in millivolt (mV) versus time in seconds. A 12-lead ECG provides twelve different perspectives of the electrical cardiac activities. In particular, leads I, AVL, V5, and V6 represent the view of the lateral wall of the heart; leads II, III, and AVF represent the inferior wall of the heart; leads V1 and V2 represent the septal wall of the heart; leads V3 and V4 represent the anterior wall of the heart; and lead AVR is used to indicate the correctness of the electrode placement. These leads can also be categorized into three different types, which are bipolar, unipolar, and precordial. Each lead is represented in various shapes of ECG morphologies. This helps cardiologists or physicians to find out where the abnormalities are.

### 3.2. ECG Morphology or ECG Waveform

**Figure 4:**
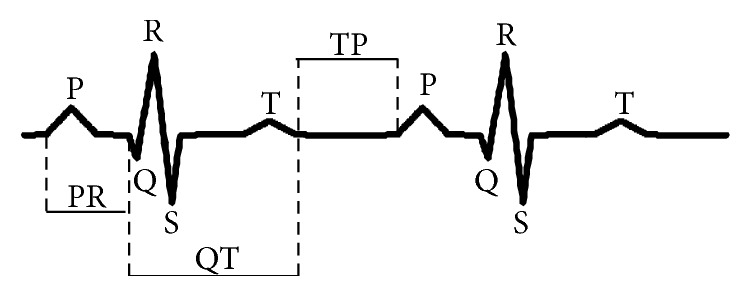
ECG morphology of two normal beats.

ECG morphology [4, 51] simply is the waveform or perspective of the electrical activity of the cardiac muscle, depolarization, and repolarization, in a cardiac cycle. The heart produces electrical impulses which spread through the cardiac muscle to make the heart contract. A normal ECG morphology consists of PQRST waves and each of the PQRST waveforms represents a single heartbeat or a cardiac cycle as shown in Figure 4.

Cardiologists use the following morphologies for diagnosis.

1. *P-wave* refers to the electrical activation of atrial depolarization that causes the conduction of electrical impulse through the atria.
2. *QRS complex* shows a ventricular depolarization which causes a contraction of the ventricles.
3. *PR-interval* starts from an onset of the P-wave to an onset of the QRS-complex.
4. *T-wave* displays the repolarization of the ventricles during the time that the ventricles return to their resting electrical state.
5. *QT-interval* starts from an onset of the QRS-complex to the end of the T-wave. QT-interval presents ventricular depolarization and repolarization.
6. *TP-segment* starts from the end of the T-wave of the previous ECG beat to the onset of the P-wave of the following ECG beat. TP-segment represents the time when the heart muscle cells are electrically silent. So, it is always illustrated by isoelectric interval which represents a zero line, a baseline, or an electric line.

### 3.3. ECG Artifacts

An ECG artifact [14, 52–55] is described as waveform interference in an ECG recording resulting from noise contamination, anything that is not caused by the electrical activity generated by the heart. Artifacts can generally be classified into 2 groups as nonphysiologic and physiologic artifacts. Nonphysiologic artifact is caused by equipment problems or interference from neighboring electrical devices, whereas physiologic artifact is caused by muscle activities or skin interferences. ECG artifact is found in various and uncertain forms, some even have complete components of ECG morphology. Four common types of ECG artifacts are shown in Table 1.

### 3.4. Dynamic Time Warping

Dynamic time warping (DTW) [57] is considered one of the most accurate similarity measures for time series data. With a dynamic programming technique to find an optimal warping path, DTW can handle nonlinear alignments or local time shifting and handle different-length subsequences [58, 59]. Consequently, in time series domain, DTW is generally more suitable than the classic Euclidean distance.

Given two time series sequences, a sequence *Q* of length *n* and a sequence *C* of length *m* are as follows:

(1) $$\begin{array}{c} Q=q_{1},q_{2},\ldots ,q_{i},\ldots ,q_{n},\\ C=c_{1},c_{2},\ldots ,c_{j},\ldots ,c_{m}. \end{array}$$

An *n*-by-*m* matrix is constructed to store the cumulative distance between any two data points, *q*_*i*_ and *c*_*j*_. The warping path can be found by dynamic programming to calculate a cumulative distance *γ*(*i*, *j*) from a sum of distance in the current cell and the minimum of the cumulative distance of the three adjacent elements as follows:

(2) γi,j=dqi,cj +min⁡γi−1,j−1,γi−1,j,γi,j−1,

where

(3) $$\begin{array}{c} d\left(q_{i},c_{j}\right)={\left(q_{i}-c_{j}\right)}^{2}. \end{array}$$

Finally, the optimal path that minimizes the warping distance is achieved and the DTW distance value is calculated as follows:

(4) $$\begin{array}{c} \text{DTW}\left(Q,C\right)=\min\left\{\sqrt{\overset{k}{\underset{k=1}{\sum }}w_{k}},\right. \end{array}$$

where *w*_*k*_ is the element (*i*, *j*)_*k*_ of the matrix and also belongs to *k*th element of a warping path *W*.

### 3.5. Contribution of Our Work

**Figure 5:**
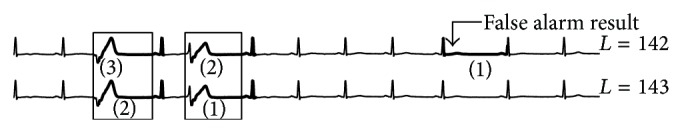
Different anomaly detection results by typical fixed-length algorithms. Only a small change in the input length *L* could produce false alarm results, detecting an extra beat as anomaly. The boxes frame real anomaly beats and the bold lines denote the results by fixed-length anomaly detection algorithm.

Our work addresses the following 4 major problems in ECG anomaly analysis.

(*1) Problem of Identifying the Length of Anomaly ECG Beat.* Each person has different cardiac cycles; in other words, each person has variable beat lengths according to individual respiratory drives. It is hard to know what the length of anomaly ECG beat will be. Unfortunately, many current algorithms are fixed-length algorithms and they require the users to specify the length themselves. As illustrated in Figure 5, a slight difference of length identification has an effect on the results of the algorithm. At *L* = 142, a typical fixed-length brute force algorithm would produce a false alarm result whereas for *L* = 143, no false alarm is detected. Therefore, the problem of identifying the beat length essentially must be addressed.

(*2) Problem of Single-Lead Consideration.* Most research on ECG mainly focuses on single-lead signal and ignores associations among other leads. However, in practice, ECG is typically recorded in many leads in order to capture activities in different perspectives. For example, for myocardial infarction (MI), so-called a heart attack, cardiologists have to consider and interpret multilead ECG to see where the lack of blood occurs in myocardium. In particular, for Inferior MI, at least, leads II, III, and AVF must all be considered. Therefore, we aim to design an algorithm to support multilead ECG and consider its association.

*(3) Problem of False Alarm Results.* Currently, many research works have emphasized on noise reduction, but none of them have proposed a method to handle the problem of ECG artifacts that mimic the ECG morphology. Additionally, existing methods may distort the ECG morphology after suppression.

Existing commercial ECG machines and applications cannot handle the false alarm problem perfectly. Bedside monitor may give excessive alarms to physicians in the case when ECG artifacts occur, interfering with perfectly normal ECG signals. This clearly wastes the physician's valuable time detecting the causes. Moreover, inexperienced physicians may misinterpret the results and give improper treatment to patients.

Consequently, our work focuses on the reduction of these false alarm results.

(*4) Problem of Re-Recording ECG.* In the case where ECG artifacts are detected, re-recording of the entire ECG from patients seems to be an easy fix. However, it could waste the physician's time in treating other patients, and it may not even find any abnormality in the re-recorded signals due to some heart diseases that contain only a few anomaly beats, for example, short run ventricular tachycardia (short run VT).

## 4. Methodology

In actual clinical practice, to differentiate a real ECG beat from an ECG artifact, cardiologists need to compare the morphology of that beat to other ECG beats in cleaner leads based on the following facts: (1) each time alignment comes from the same electrical activity across every lead and (2) TP segment is an isoelectric interval on the ECG, which should always be at the baseline; that is, any changes in TP segment must be considered as ECG artifacts. It is noted that U-wave is not considered in this work because it is just a condition that does not properly reflect anomalous ECGs, such that the physician needs further clinical investigation and diagnosis such as potassium level in the blood.

It is generally difficult to identify a TP segment in ECG artifacts. Therefore, we propose a method to first locate TP segments in the least contaminated ECG and to use it as a reference to identify TP segments in other leads.

We propose a robust and accurate anomaly detection algorithm in ECG artifacts (RAAD) by applying clinical knowledge from cardiologists and techniques from time series mining. The algorithm consists of preprocessing step, cleanest lead discovery, morphology segmentation, and robust anomaly detection.

### 4.1. Preprocessing

Generally, the frequency of baseline wandering is less than 0.5 Hz, and AC interference is in the range of 50–60 Hz [60–63]. To suppress the baseline wandering and AC interference, we apply second-order Butterworth [63], zero-phase digital filtering [62] in the preprocessing step through functions available in MATLAB. We have conducted extensive preliminary experiments to determine the proper cut-off frequency for band-pass filter from a wide range of frequency from 0.5 to 50 Hz. As a result, a low-pass filter at 20 Hz is used to reduce the AC Interference, and then a high-pass filter at 2 Hz is used to reduce the baseline wandering because the frequency spectrum at these ranges has been shown not to distort the ECG morphology for PQRST detection and ECG interpretation.

### 4.2. Cleanest Lead Discovery

Ideally, a clean lead is a lead that is not contaminated by noise. However, in reality where noise is inevitable, the cleanest ECG lead is considered the lead that is least contaminated. The shape of ECG beats within the same lead are generally similar to each other. So, if the shape of an ECG beat is different, we can suspect it to be an abnormality or an artifact.

Therefore, we assume that the lead that has the highest number of similar ECG beats is the cleanest lead. This corresponds to the definition of motif in time series mining task, which defines a motif as a group of frequently occurring patterns.

In our work, proper length motif discovery algorithm [38] is utilized to identify the cleanest lead that produces the maximum frequency of motif. In addition, an anomaly candidate in the cleanest lead is obtained from the remaining beats that are excluded from the motifs.

This algorithm is based on a minimum description length [64] which is a parameter-free algorithm and uses the* bitsave* as a heuristic to obtain the set of motif. The higher the score of* bitsave* is, the more similarity there is of patterns in a lead. Consequently, it is used to indicate the cleanest lead. We further extend the algorithm in [38], as follows.

(1) The starting length is instead determined by a sampling rate and expert knowledge. The sampling rate is a number of ECG samples or data points per second. In view of expert knowledge [3, 65, 66], the length of systole is 1/3 of the cardiac cycle and the length of diastole is 2/3 of the cardiac cycle at resting. In addition, the normal heart rate range is 60 to 100 beats per minute. Therefore, the starting length is calculated as follows:
  (5) $$\begin{array}{c} \text{starting}\;\text{length}=\frac{1}{3}\times \frac{100}{60}\times \text{sampling}\;\text{rate}. \end{array}$$
(2) To find a motif candidate, instead of using the *k*th-compression motif as a motif candidate as in [38], our algorithm uses only the 1st compression motif which is the most similar pair of subsequences.
(3) We use only the first motif as the result in each ECG lead. The first motif consists of a motif candidate, a pair of the most similar subsequences, and its neighboring subsequences which are similar to the motif candidate.

The algorithm is summarized as in Algorithm 1. More details are available in [38].

Lines 1-2: Extract all ECG* subsequences C* from ECG *T* with starting position *i*. Formally, *C*_*i*_ = *t*_*i*_, *t*_*i*+1_,…, *t*_*i*+*m*−1_ for 1 ≤ *i* ≤ *n* − *m* + 1, where *n* is the length of ECG and *m* is the length of subsequence.

Line 3: Z-normalization: to address the problems of amplitude scaling and offset translation in subsequences, all data points in subsequence are normalized as follows:

(6) $$\begin{array}{c} {\hat{t}}_{j}=\frac{t_{j}-\text{mean}\left(C_{i}\right)}{std\left(C_{i}\right)}, \end{array}$$

where ${\hat{t}}_{j}$ is the normalized value of the *j*th data point in subsequence *C*_*i*_. Therefore, the result for *C*_*i*_ is a set of real number sequences ${\hat{t}}_{j},{\hat{t}}_{j+1},\ldots ,{\hat{t}}_{j+m-1}$, where *i* ≤ *j* ≤ *n* − *m* + 1.

Lines 4–6: The most similar pair of subsequences *A*, *B* are identified as motif candidates with the lowest Euclidean distance and then* bitsave* of the pair is computed by createGroup function. The center is calculated from the average of *A* and *B*.

Lines 7–14: Find a neighboring subsequence, and then compute new bitsave as *bs*. If *bs* is higher than the group's* bitsave*, the neighboring subsequence is added to the group. This step is repeated until the new *bs* cannot improve any further. At this point, we obtain a group of similar subsequences at length *n* in a single lead.

Line 15: The group which is updated to be a motif must have higher* bitsave*.

Next, the algorithm repeats lines 1–16 for every possible length. Finally, the result of the motif discovery in a lead is the group that has maximum* bitsave*.

The abovementioned algorithm runs on a single lead. So, in finding the cleanest lead, the algorithm must also run on every single lead. The lead that has the maximum number of* bitsave* becomes the cleanest lead.

### 4.3. Morphology Segmentation

The morphology segmentation aims to specify TP segments in ECG artifacts by referring to the position of PQRST in the cleanest lead. To identify the position of PQRST, we first locate an R-peak which is a striking peak, then a P-wave which is a waveform before the R-peak, and then a T-wave which is a waveform after the R-peak.

Our work uses difference operation method (DOM) [67] to find QRS complex. DOM is selected because it is not complicated and has been applied in several works [60, 68, 69]. We slightly modify DOM to locate the PQRST waveform, incorporating the expert knowledge [3, 70] about normal ECG waveform to justify thresholds as shown in Table 2.

The algorithm is presented as follows.

**Step 1 .**

Extract a QRS complex using DOM. In this step, we acquire the positions of Q, R, and S.

**Step 2 .**

Extract a P-wave by first finding the P point as the maximum voltage before the Q point within 0.20 second (0.20 second is an upper bound of a normal PR interval). Afterwards, we retrieve a starting point and an end point of the P-wave by finding the minimum voltage position before and after the P point within 0.06 second (0.06 second is half of the upper bound of a normal P-wave). In this step, we acquire the onset, the peak, and the end of the P-wave.

**Step 3 .**

Extract a T-wave by first finding a T point as the maximum voltage after an S point within 0.38 second (0.38 second is the difference between an upper bound of a normal QT interval and a lower bound of a normal QRS complex). Afterwards, we retrieve a starting point and an end point of the T-wave by finding the minimum voltage position before and after the T point within 0.06 second. In this step, we acquire the onset, the peak, and the end position of the T-wave.

After we apply steps 1–3 to the ECG in the cleanest lead, a TP segment is identified as a period from the end of the T-wave to the onset of the P-wave of the next cycle. Finally, we can use all the TP segments in the cleanest lead as references to the TP segments in other leads.

### 4.4. Robust Anomaly Detection

We use motif discovery algorithm to identify a period of a motif and a period of anomaly candidates. A period of motif can group normal beats in the cleanest lead and also can give some hints of any artifacts, anomaly, or normal beats in other leads. In this step, dissimilar beats from normal beats are considered as anomalies. A period of anomaly candidates is the rest of the ECG subsequence that is not detected as a motif. In this step, the period of anomaly candidates is then shifted accordingly to align with a cardiac cycle. Finally, these two periods are produced as the result of the algorithm. The challenges of this stage are listed below.

1. Subsequence extraction technique: each subsequence is extracted from the whole ECG, starting from the onset position of a P-wave to the end of a T-wave. Therefore, each subsequence represents each ECG beat according to actual cardiac cycle.
2. Partial similarity calculation: to measure the dissimilarity between two variable-length subsequences, dynamic time warping (DTW) is useful because of its nonlinear alignment handling abilities. However, using DTW can cause a problem of excessive alignment such as a P-wave aligning with a QRS complex. Therefore, we propose a new method that limits the alignment within each portion of ECG morphology. The algorithm calculates DTW distance only between the two-beat pair at each portion of morphology, that is, P-wave, PR interval, and QT interval.

The algorithm is presented in Algorithm 2.

Inputs of the algorithm are an ECG in each lead, the positions identified by the morphology segmentation step and the anomaly candidates obtained from motif discovery algorithm.

Lines 1–5: The P-wave, PR interval, and QT interval are extracted from the period of the motif.

Lines 6–18:* nearestneighbordis* of beat *p* is the shortest distance between the beat *p* and other beats.

Lines 10–12: Distance calculation between beats *p* and *q* is computed; the distance is the total summation of DTW distance of the P-wave, PR segment, and QT interval.

Line 19: The* newanomaly* is the beat that has a larger* nearestneighbordis* distance than the threshold. The threshold is set to be a sum of the mean and the standard deviation of distances. Since most beats are similar to each other, the distances are not varied much. Therefore, dissimilar beats will be considered anomaly beats.

Lines 20-21: The starting position and the end position of anomaly candidates are shifted to the closest *ps* and *te*, respectively. The* anomalybeats* is the result of our work. It is from a collection of the* newanomaly* and nonmotif beats from the motif discovery step.

## 5. Experiments

### 5.1. Experimental Setup

#### 5.1.1. Real ECGs

We conducted experiments on real ECG datasets taken from PhysioNet [71] as shown in Table 3. A variety of datasets are used to illustrate various cases of comparison, that is, anomaly detection with normal ECG, single-lead ECG, multilead ECG, and various ECG artifacts.

**Figure 6:**
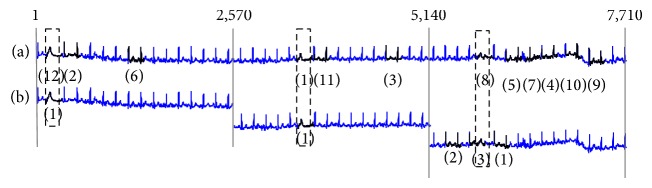
Comparison of existing algorithm on two different settings in the number of data points used. (a) The whole 7,710 data points were run at one time and (b) the signal was split into separate runs, each with 2,570 data points. The dashed-line box frames the period of real anomaly ECG beat. The number in parentheses identifies the order of detected anomaly subsequence from existing algorithm. It is apparent that more false alarms are detected when longer ECG is used in the calculation.

We also perform some empirical studies and analysis to find out whether a number of data points have any effect on the result of the existing algorithms. As shown in Figure 6, we found that the more data points used in the calculation, the more false alarm results are generally produced because the existing algorithms consider similar anomaly beats that occur more than once so that the dissimilarity is low. On the other hand, our work considers the number of anomaly beat occurrences, so that the number of data points has no effect on the result. To be fair to other methods, we used less than 3,600 data points/lead, which is the typical length that can fit nicely on printouts of a standard 12-lead ECG and computer screen display.

#### 5.1.2. Competitive Algorithms

One of the most recent anomaly detection algorithms for time series sequences and two of the most popular algorithms, BitClusterDiscord [36], brute force discord discovery (BFDD) algorithm [21] and HOT SAX algorithm [35], have been chosen to be compared with our proposed work. BFDD produces exact anomaly subsequences whereas HOT SAX and BitClusterDiscord produce an approximate anomaly subsequence while avoiding noise inference through the discretization method and bit serialization, respectively. However, these algorithms require the length of anomaly beat (*L*) as a predefined input. To give the best advantage to these three rival methods, we provide them with the precise lengths of actual anomaly beats *L* specified by cardiologists as shown in Table 3.

#### 5.1.3. Evaluation Metrics

The algorithm is evaluated based on 5 measurements: AoD (accuracy-on-detection) [72], sensitivity, specificity, positive predictive value, and false alarm rate. Before stating how to evaluate the detected anomaly subsequence, the overlap criteria must be explained.

(*1) Overlap between the Detected Anomaly Subsequence and the Real Anomaly Beat.* Evaluation based on overlaps is extremely subjective. How much of the overlap with the ground truth anomalous beat would be enough to be considered a correct detection? To give an extensive evaluation, we define various criteria for the overlaps as follows.

(*i) Overlap Based on Thresholds.* The detection result is considered correct when its overlapping ratio with the ground truth is greater than a specified threshold. In our experiments, the thresholds are set at 0%, 30%, 40%, and 80% due to the following supportive reasons.

- 0% is most accommodating; even with only one overlapped data point, the result is considered a correct detection.
- 30% and 40% are a little more restricted. These numbers are approximate percentages of data points typically covering an anomalous beat's morphology.
- 80% is much tighter and has been used in some research work [73].

We define an* overlapping ratio* as a ratio between a number of the detected data points *D* that overlaps the ground truth anomalous beats *R* and the length of that ground truth anomalous beat. It can be calculated as follows:

(7) $$\begin{array}{c} \text{Overlapping}\;\text{ratio}\left(R,D\right)=\frac{\left|R\cap D\right|}{\left|R\right|}\times 100, \end{array}$$

where *D* is the set of the resulting data points from the algorithm and *R* is the set of the ground truth data points according to cardiologist's analysis.

(*ii) Overlap Based on Clinical Diagnosis by Cardiologists.* In clinical diagnosis process, a cardiologist needs to analyze the anomaly detection results produced by the algorithm or ECG machines by examining the morphology of those signals to make a final diagnosis. Ideally, the results that only highlight the crucial morphology would be very beneficial. Therefore, the overlap between a detected subsequence and a real anomaly beat should correspond to two conditions as follows.

**Figure 7:**
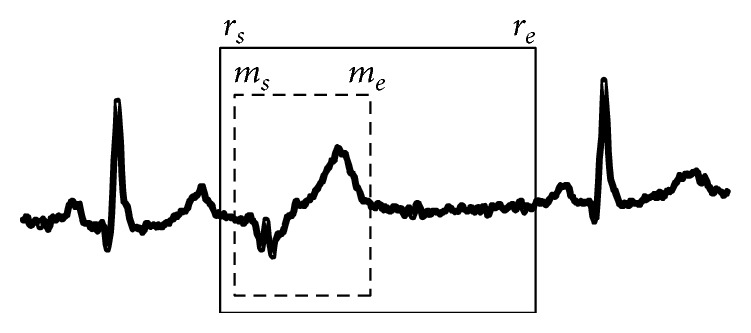
Actual anomaly occurs within a dashed box, whereas the complete morphology of one cardiac cycle is covered in a solid box.

- The overlap must cover the area of morphology that is between the starting point *m*_*s*_ and the end point *m*_*e*_ of the morphology of anomaly beat, as shown in Figure 7.
- The overlap must* not* cover any adjacent beats. It means the algorithm can produce results that are, in fact, longer than those in (a) and must be contained within the anomaly beat which is between the starting point *r*_*s*_ and the end point *r*_*e*_. *r*_*s*_ is an end point of a T-wave of the previous cardiac cycle and *r*_*e*_ is a starting point of a P-wave of the following cardiac cycle, as shown in Figure 7.

(*2) Accuracy on Detection (AoD).* AoD [72] is used to indicate how well an algorithm can recognize anomalous ECG beats. AoD refers to an average percentage of detected subsequences that cover real anomaly beats. It is important to note that the higher the AoD, the better the accuracy of the detection. AoD can be calculated as follows:

(8) $$\begin{array}{c} \text{AoD}=\frac{\sum_{i=1}^{K}\text{Ovelapping}\;\text{ratio}\left(R_{i},D_{i}\right)}{K}\times 100, \end{array}$$

where *K* denotes the number of real anomaly beats. *R*_*i*_ denotes a real anomaly beat. *D*_*i*_ denotes a detected subsequence that overlaps with the real anomaly beat *R*_*i*_.

(*3) False Alarm Rate.* False alarm rate or false positive rate refers to a percentage of normal beats that are incorrectly detected by an algorithm, as they are instead identified as anomalous. It can be calculated as follows:

(9) $$\begin{array}{c} \text{False}\;\text{alarm}\;\text{rate}=\frac{\text{False}\;\text{Positive}}{\left(\text{False}\;\text{Positive}+\text{True}\;\text{Negative}\right)}\times 100, \end{array}$$

where a true negative denotes the number of normal ECG beats that are correctly identified as normal and a false positive denotes the number of normal ECG beats that are incorrectly identified as anomalous as shown in Table 4.

(*4) Sensitivity or Recall.* Sensitivity refers to a percentage of real anomalous beats that are correctly detected by an algorithm. It can be calculated as follows:

(10) $$\begin{array}{c} \text{Sensitivity}=\frac{\text{True}\;\text{Positive}}{\left(\text{True}\;\text{Positive}+\text{False}\;\text{Negative}\right)}\times 100, \end{array}$$

where a true positive denotes the number of real anomalous beats that are correctly identified as anomalous and a false negative denotes the number of real anomalous beats that are incorrectly identified as normal, that is, missing the abnormalities of those beats in the detection.

(*5) Specificity.* Specificity refers to a percentage of real normal beats which are correctly identified by an algorithm. It can be calculated as follows:

(11) $$\begin{array}{c} \text{Specificity}=\frac{\text{True}\;\text{Negative}}{\left(\text{True}\;\text{Negative}+\text{False}\;\text{Positive}\right)}\times 100. \end{array}$$

(*6) Positive Predictive Value (PPV) or Precision.* Positive predictive value (PPV) refers to a proportion of the correctly identified anomalous beats within the entire set of anomalous detection results. PPV can be calculated as follows:

(12) $$\begin{array}{c} \text{PPV}=\frac{\text{True}\;\text{Positive}}{\text{True}\;\text{Postive}+\text{False}\;\text{Positive}}\times 100. \end{array}$$

### 5.2. Experimental Results

#### 5.2.1. Anomaly Detection Results with Normal ECG

**Figure 8:**
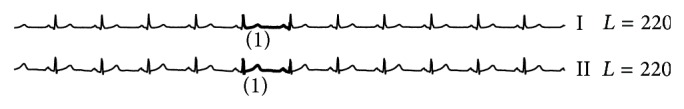
The false alarm results from BFDD on the INCARTDB01 dataset. The number in parentheses identifies the order of detected anomalies.

**Figure 9:**
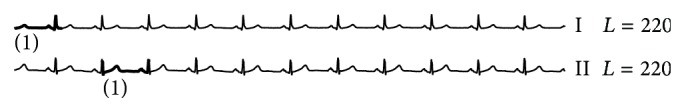
The false alarm results from HOT SAX on the INCARTDB01 dataset.

**Figure 10:**
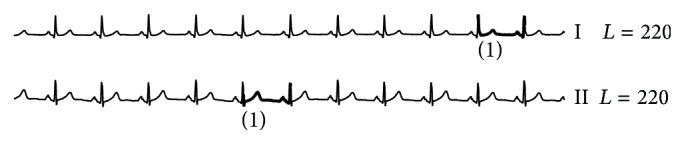
The false alarm results from BitClusterDiscord on the INCARTDB01 dataset.

On the INCARTDB01 dataset which only contains normal ECG signals, RAAD works correctly as it produces no anomaly beat. On the other hand, other algorithms did produce anomaly beat results as shown in Figures 8, 9, and 10. Due to space limitations, we only show two ECG leads; the full detail is provided on our support website [74]. AoD, sensitivity, and positive predictive value are not calculated since no anomaly beats are present. However, the experiment results evidently show that RAAD outperforms other competitive algorithms in terms of both specificity and false alarm rate.

#### 5.2.2. Anomaly Detection Results with Single-Lead ECG

**Figure 11:**
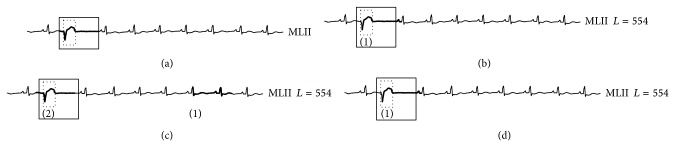
Anomaly detection results among three algorithms on MITDB dataset. (a), (b), (c), and (d) are results of our proposed RAAD, BFDD, HOT SAX, and BitClusterDiscord, respectively. RAAD produced correct results whereas BFDD, HOT SAX, and BitClusterDiscord produced incomplete results along with some false alarm.

**Figure 12:**
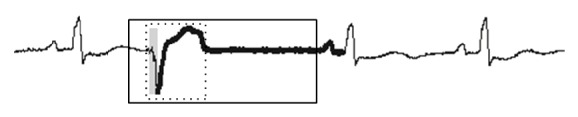
A zoom-in picture of the anomaly subsequence in Figure 11(b). Some part of the anomaly's morphology is missing from the detection, and some part of the following beat is covered.

Many existing algorithms can only detect anomalies in one single lead. This experiment therefore aims to compare effectiveness among existing works and our proposed RAAD algorithm on a single-lead ECG (MITDB dataset). Even though RAAD is designed under multilead setting, Figure 11 demonstrates that RAAD can correctly detect premature ventricular contraction (PVC) in accordance with cardiologists' diagnosis and has superior performance to other competitive algorithms because the result from RAAD covers an entire morphology of PVC as shown in a dotted-line box in Figure 11(a) and also does not cover any portion of adjacent beats as shown in a solid-line box in Figure 11(a). More importantly, no false alarm results are produced. On the other hand, BFDD and BitClusterDiscord detect an anomaly subsequence that does not completely cover the morphology of the anomalous beat, and they cover some portion of the following beat, as shown in the dotted-line and solid-line boxes of Figures 11(b) and 11(d) and the zoom-in picture in Figure 12. In HOT SAX algorithm, its first detection turns out to be a false alarm (shown as (1) in Figure 11(c)), and the second detection does not cover the entire beat; that is, some portion of the previous beat is covered, but some part of TP segment at the end of the beat is missing (shown as (2) in Figure 11(c)).

Therefore, the result of our proposed RAAD can be instantly utilized for clinical diagnosis because RAAD can obtain the result that corresponds to the cardiologists' diagnosis.

Table 5 is provided to compare the results of AoD, sensitivity, positive predictive value, specificity, and false alarm rate with several overlap criteria. We use Car. as an abbreviation of cardiologists' diagnosis criterion. In particular, for rival methods, only the results for 40%, 80%, and cardiologists' diagnosis overlap criteria are shown because the results of 0% and 30% overlaps are identical to those of 40%. Likewise, for RAAD, only the results for 80% and cardiologists' diagnosis overlap criteria are shown because the results of 0%, 30%, and 40% overlaps are identical to those of 80%. The complete results are provided in our support website [74].

As evidently shown in Tables 5 and 6, RAAD's results are quite promising as its AoD is nearly 100%; sensitivity, positive predictive value, and specificity are 100%, and false alarm rate is 0%. Overall results demonstrate that RAAD can correctly detect anomaly beats in a single-lead ECG in accordance with all criteria and measurements. No false alarm results were produced in this case.

#### 5.2.3. Anomaly Detection Results with Multilead ECG Containing ECG Artifacts and Variable Length of Anomaly Beat

ITDB, INCARTDB02, and INCARTDB03 datasets are used in these experiments, as they contain multiple-lead signals (2–12 leads), with various lengths. Tables 5 and 6 show the results of AoD, sensitivity, positive predictive value, specificity, and false alarm rate with several overlap criteria. Since the datasets contain multiple signals, to give the best advantage to the rival methods, we tested the algorithm on each lead independently given the exact lengths of the anomalous beats (cf. Table 3) and then reported the best result among all the ECG leads, along with the mean (*μ*) and standard deviation (SD). However, as our proposed RAAD can handle multilead data, one single result is produced.

**Figure 13:**
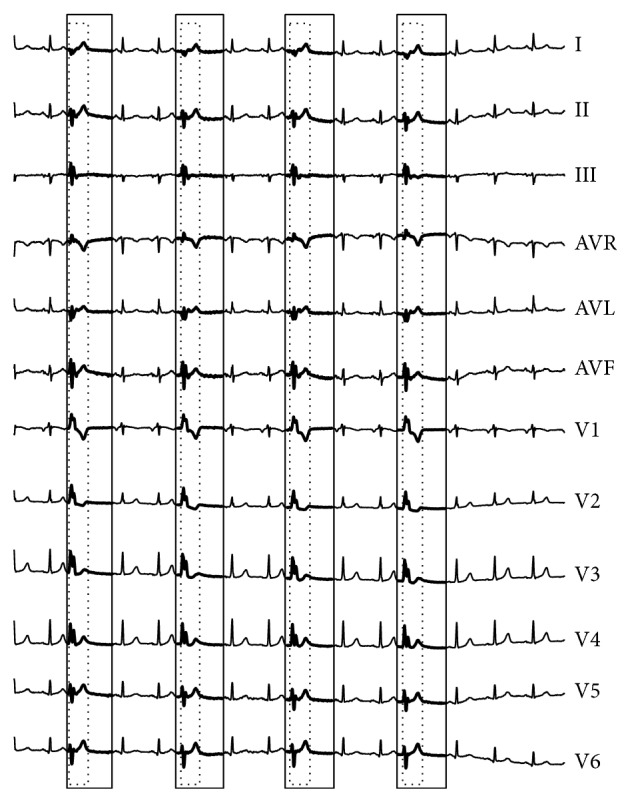
Anomaly detection results of the INCARTDB03 dataset by our proposed RAAD algorithm.

**Figure 14:**
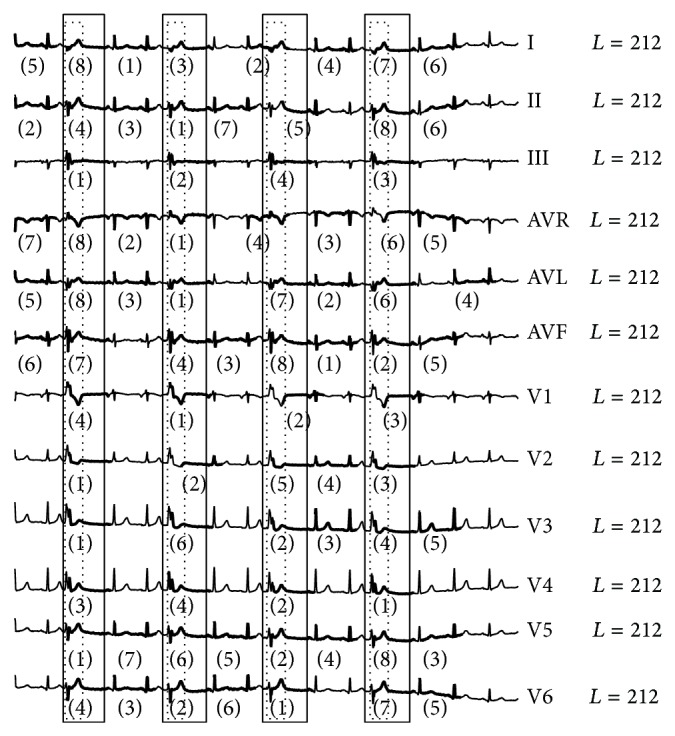
Anomaly detection results of the INCARTDB03 dataset by BFDD algorithm with *L* = 212.

Due to space limitations, we only show the result of the INCARTDB03 dataset for RAAD in Figure 13 and BFDD in Figure 14. The results of HOT SAX and BitClusterDiscord are identical to those of BFDD, so it is not presented.

In Figure 13, RAAD does not produce any false alarm results in any lead and can give the same anomaly detection subsequences as cardiologists' diagnosis even if the lengths of anomaly beats are varied. The result of RAAD covers an entire morphology of PVC (trigeminy) as shown in the dotted-line boxes and also does not cover any portion of adjacent beats as shown in the solid-line boxes. On the other hand, in Figure 14, BFDD produces many false alarm results. Although the algorithm can detect anomaly subsequences, these results do not cover the entire anomalous beats.

We would like to reemphasize that the results of our proposed RAAD can be instantly utilized for clinical diagnosis because RAAD can obtain the results that correspond to actual diagnosis by cardiologists.

The results confirm that our proposed RAAD is able to accurately detect anomaly beats in multilead ECG in accordance with cardiologists' diagnosis even when the ECG is contaminated with artifacts. Additionally RAAD can efficiently identify anomalous beats with variable lengths. In ITDB, INCARTDB02 and INCARTDB03 datasets and AoDs are nearly 100%; sensitivity, positive predictive value, and specificity are 100%, and false alarm rates are 0%. Standard deviations (SD) are always 0 due to identical results in all leads.

#### 5.2.4. Anomaly Detection Results with Multilead ECG Containing ECG Artifacts That Mimic ECG Morphology

**Figure 15:**
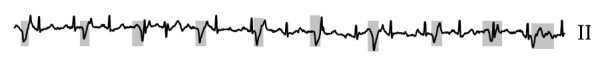
A sample of lead II in the INCARTDB04 dataset. The shaded areas show the ECG artifacts which are similar to the ECG morphology.

The INCARTDB04 dataset contains ECG artifacts that mimic the shape of the ECG morphology as shown in the shaded areas in Figure 15. The experiment was conducted on a 12-lead ECG. Due to space limitations, only the results of lead I and lead II are shown. The results of other leads are identical to those of lead II.

**Figure 16:**
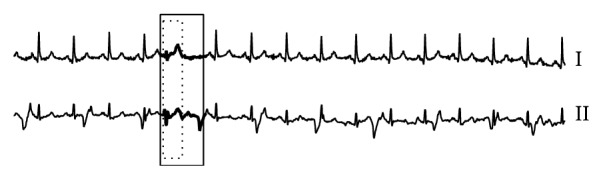
Anomaly detection result of the INCARTDB04 dataset by our proposed RAAD algorithm.

In Figure 16, RAAD does not produce any false alarms in any lead and can obtain the same anomaly detection results as those obtained by cardiologists' diagnosis even if the ECG is contaminated by noise. The results by RAAD can still cover the entire morphology of PVC as shown in a dotted-line box and also does not cover any portion of adjacent beats with the detected subsequence all contained within the solid-line box.

**Figure 17:**
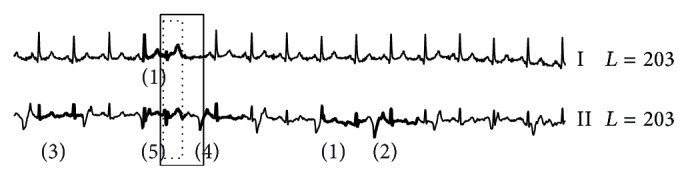
Anomaly detection of the INCARTDB04 by BFDD with *L* = 203.

**Figure 18:**
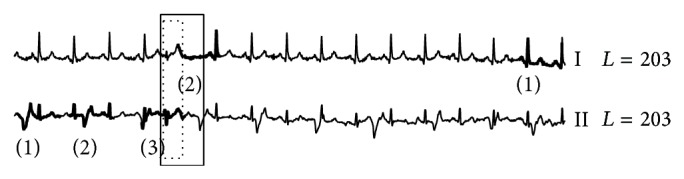
Anomaly detection result of the INCARTDB04 dataset by HOT SAX algorithm with *L* = 203.

**Figure 19:**
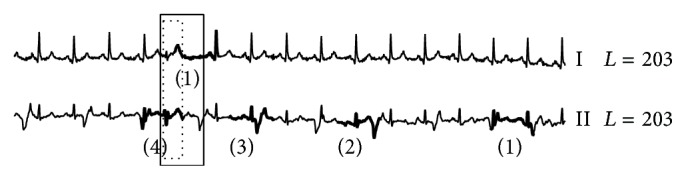
Anomaly detection result of the INCARTDB04 dataset by BitClusterDiscord algorithm with *L* = 203.

On the other hand, BFDD, HOT SAX, and BitClusterDiscord did produce various false alarm results, as shown in Figures 17, 18, and 19. Although the algorithm can still detect anomalous subsequences, they do not properly cover entire anomalous beats.

It is clearly shown that the results by our proposed RAAD can be instantly utilized for clinical diagnosis because RAAD can obtain the results that correspond to actual cardiologists' diagnosis.

Tables 5 and 6 show that RAAD is able to accurately detect the anomaly beat in multilead ECG in accordance with cardiologists' diagnosis even when the ECG contains artifacts that mimic ECG morphology. In INCARTDB04 datasets, AoDs are nearly 100%; sensitivity, positive predictive value, and specificity are 100%, and false alarm rates are 0%. Standard deviations (SD) are always 0 due to identical results in all leads.

#### 5.2.5. Anomaly Detection Results with Multilead ECG Containing Extremely Noisy ECG Artifacts That Mimic ECG Morphology

**Figure 20:**
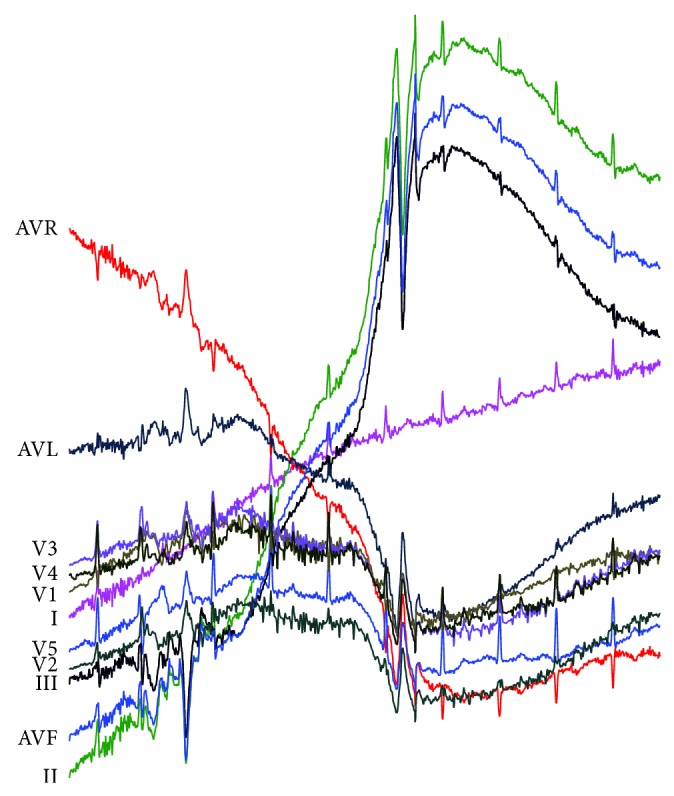
INCARTDB05 Dataset.

The INCARTDB05 dataset contains very noisy ECG artifacts as shown in Figure 20.

**Figure 21:**
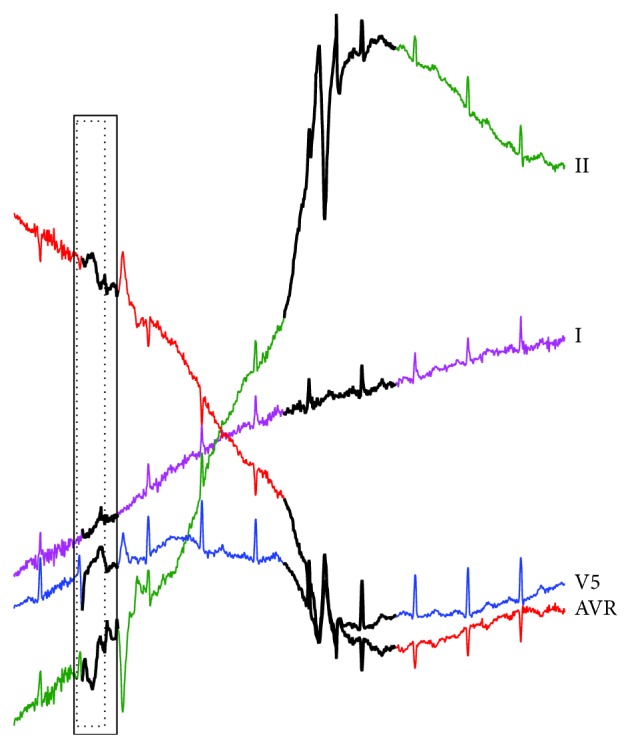
Anomaly detection results for the INCARTDB05 dataset by RAAD algorithm.

**Figure 22:**
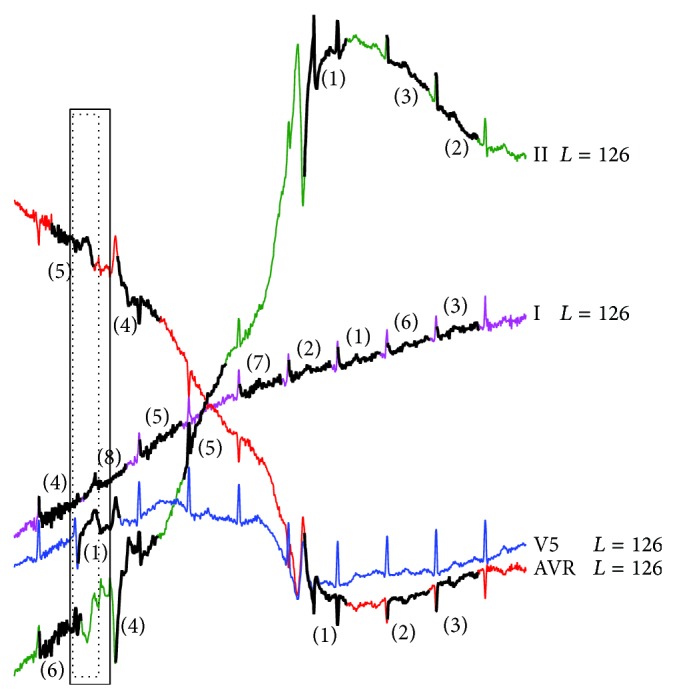
Anomaly detection results for the INCARTDB05 dataset by BFDD algorithm with *L* = 126.

Figures 21 and 22 are shown to compare the results of RAAD and BFDD. Due to space limitations and full clarity/readability in the figures, only the results of leads I, II, AVR, and V5 are shown. The results of other leads are very similar. The results of HOT SAX and BitClusterDiscord are also similar to that of BFDD so they are not presented here. Therefore, the complete results and details are provided in our support website [74]. The results show that all algorithms are able to detect the real anomaly beat. However, RAAD produces much fewer false alarm results than BFDD, HOT SAX, and BitClusterDiscord. Nonetheless, the results of all four algorithms still do not cover the morphology which is essential for diagnosis.

According to Tables 5 and 6 with the INCARTDB05 dataset, the overall results from RAAD cover the portion of real anomaly beat more than those from competitive algorithms, as indicated by mean AoD results.

According to this failure, it suggests that RAAD may not be appropriate for very noisy ECG artifacts because it is difficult to detect PQRST morphology. Likewise, it is difficult for other algorithms or even for nonexperienced physicians to interpret and detect anomaly beats accurately.

## 6. Discussion

According to all experiments, overall results indicate that RAAD outperforms the other existing algorithm and can be used for both single-lead and multilead ECGs. Additionally, variable lengths in anomaly beats have no effect on RAAD and no predefined length of anomaly beat is required. It is because our algorithm relies on ECG morphology analysis that cardiologists use for diagnosis in clinical practice, together with a utilization of the proper-length motif discovery technique.

When compared with the competitive algorithms, RAAD outperforms others because the detected subsequences do cover real anomaly beats accurately and correspond to cardiologists' diagnosis. Consequently, the result of RAAD can be promptly utilized by cardiologists.

In addition, to overcome the problem of ECG artifacts that mimic ECG morphology, RAAD considers the association of each lead so the algorithm uses the cleanest lead as a reference lead to help identify ECG artifact that mimic ECG morphology. On the other hand, the competitive algorithms produce numerous false alarm results because they consider each lead independently and do not utilize expert knowledge.

With RAAD's overall sensitivity of 100%, it is shown that our algorithm can discover all anomaly beats and does not generate any false negatives. Moreover, with 0% and much smaller number of false alarm rates, it is shown that our algorithm can significantly reduce false alarm results. However, our algorithm still has a limitation when applied to extremely noisy ECG artifacts as it is very difficult to accurately detect anomaly beat. Likewise, this also happens to other algorithms as well as to the cardiologists themselves. The last dataset in Section 5.2.5 is taken directly from the Physionet repository, where no specific cause such very noisy ECG signal was given. Nonetheless, in view of cardiologists, they would not use such a noisy ECG for diagnosis and assume that such a very noisy ECG signal may be recorded from a patient who has tremor or agitation or myoclonus. To address this problem, the patient would be immobilized before performing an ECG re-recording or another investigation might alternatively be considered. It is apparent that the validation for such case is very difficult since all of the evaluation and validation for ECG anomaly detection problems are based solely on the expert's opinion/diagnosis; we would have no ground truth for the problem if the cardiologist is unable to annotate the signals. As a future work to alleviate this difficulty, an improvement of ECG artifact reduction/removal algorithms should be done, making the results more trustworthy to the cardiologists.

## 7. Conclusions

This research proposes a novel algorithm for robust and accurate anomaly detection in ECG artifacts. Motif discovery is used to find normal beats and identify the cleanest lead. The cleanest lead is utilized to detect positions of PQRST on the lead and other leads. ECG morphology is used to compare the similarity of each beat instead of a calculation of the whole subsequences like other algorithms.

The experimental results reveal that our proposed RAAD yields better anomaly detection results than brute force algorithm (BFDD), HOT SAX algorithm, and BitClusterDiscord algorithm. The results of RAAD cover the morphology that is essentially used for actual diagnosis and can significantly reduce false alarm rates. In the meantime, it can be used for both single-lead and multilead ECG; no predefined length of anomaly beat is required, and it can be applied with ECG artifacts even when they mimic ECG morphology. Finally, the result of RAAD can promptly be utilized by cardiologists. In the future, we will improve the algorithm to support real-time detection.

## Figures and Tables

**Algorithm 1:**
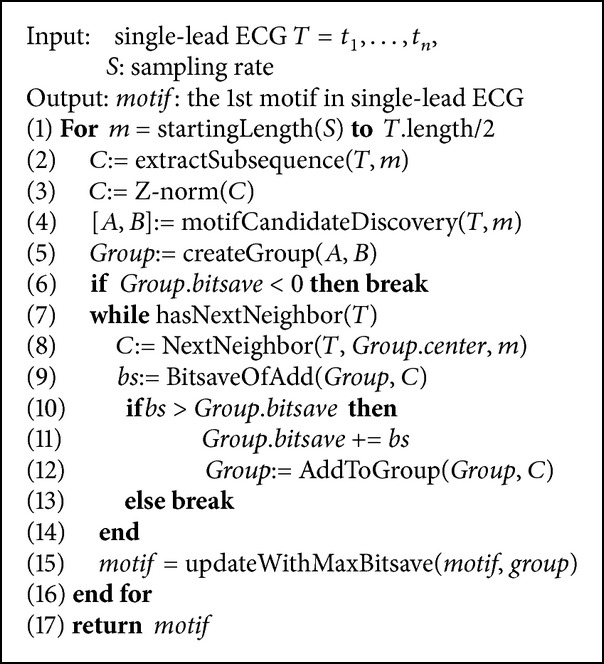
Proper length motif discovery algorithm for ECG.

**Algorithm 2:**
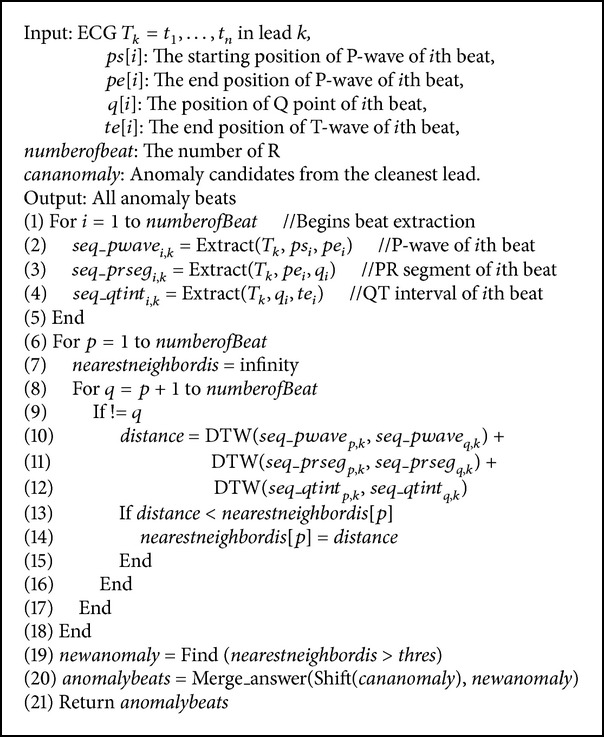
Robust anomaly detection.

**Table 1:** Common ECG artifacts with description, causes, and example.

Artifacts	Description	Cause of artifact	Example
(1) Wandering baseline	A slow wander of the baseline	(i) Body movement (ii) Respiratory swing	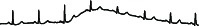

(2) AC interference	Varying amplitude of ECG and indistinct isoelectric baseline	(i) Electrical power Leakage (ii) Improper equipment grounding (iii) Close proximity of other electrical equipment	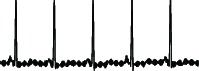

(3) Muscle tremor	Narrow and rapid spike of ECG	(i) Effect of EMG signal (ii) Shivering (iii) Parkinson's disease	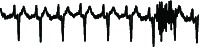

(4) Motion artifact	Large swing in the baseline, uncertainty of large amplitude signals	(i) Effect of epidermal signal (ii) Stretching the epidermis (iii) Coughing (iv) Ambulation	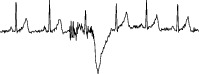 [56]

**Table 2:** List of normal ranges.

Part of waveform	Normal range
PR interval	0.12–0.20 sec.
P-wave	≤0.12 sec.
QRS complex	0.06–0.10 sec.
QT interval	0.36–0.44 sec.

**Table 3:** Summarized details of datasets obtained from the Physionet archive [37].

Dataset	Database	Number of leads	Length of anomaly beat (*L*)	Number of data points per lead	Description	Artifact
(1) INCARTDB01	The St. Petersburg Institute of Cardiological Technics 12-lead Arrhythmia Database (record I02 from 00.27.40 to 00.27.50)	11 leads: I, II, III, AVR, AVL, AVF, V1, V2, V3, V4, V5	No anomaly	2,570	(i) ECGs only consist of normal ECG beats. (ii) Length of normal beat of almost all beats is 220.	No

(2) MITDB	The MIT-BIH Arrhythmia Database (record 108 from 00.09.30 to 00.09.40)	1 lead: MLII	554	3,600	(i) Contains one anomalous beat of premature ventricular contraction.	No

(3) ITDB	The MIT-BIH Long Term Database (record 14046 from 01.41.10 to 01.41.20)	2 leads: ECG1, ECG2	146 154	1,280	(i) Contains two anomaly ECG beats.	Yes

(4) INCARTDB02	The St. Petersburg Institute of Cardiological Technics 12-lead Arrhythmia Database (record I01 from 00.01.40 to 00.01.50)	12 leads: I, II, III, AVR, AVL, AVF, V1, V2, V3, V4, V5, V6	203 217 223	2,570	(i) Contains three anomalous beats with a variety of ECG artifacts present in all leads.	Yes

(5) INCARTDB03	The St. Petersburg Institute of Cardiological Technics 12-lead Arrhythmia Database (record I01 from 00.15.30 to 00.15.40)	12 leads: I, II, III, AVR, AVL, AVF, V1, V2, V3, V4, V5, V6	212 223 223 234	2,570	(i) Contains four anomalous ECG beats of premature ventricular contraction (Trigeminy) with various artifacts present in all leads.	Yes

(6) INCARTDB04	The St. Petersburg Institute of Cardiological Technics 12-lead Arrhythmia Database (record I01 from 00.00.00 to 00.00.10)	12 leads: I, II, III, AVR, AVL, AVF, V1, V2, V3, V4, V5, V6	203	2,570	(i) Contains one anomalous beat of ventricular ectopic with various artifacts present in all leads.	Yes

(7) INCARTDB05	The St. Petersburg Institute of Cardiological Technics 12-lead Arrhythmia Database (record I02 from 00.09.34 to 00.09.40)	11 leads: I, II, III, AVR, AVL, AVF, V1, V2, V3, V4, V5	126	1,531	(i) Contains one anomalous beat with very noisy artifacts in all beats.	Extremely noisy ECG artifacts

**Table 4:** Interpretation of true positive, false positive, false negative, and true negative.

Detection	Diagnosis	
	Anomaly beat	Normal beat
Anomaly beat	True Positive	False Positive
Normal beat	False Negative	True Negative

**Table 5:** The best value, mean (*μ*) and standard deviation (SD) of AoD, sensitivity, positive predictive value, specificity, and false alarm results obtained by BFDD, HOT SAX, and RAAD with various overlap criteria. Bold figures denote the wining algorithms.

	AoD								Sensitivity								Positive predictive value								Specificity								False alarm rate							
	BFDD			HOT SAX			RAAD		BFDD			HOT SAX			RAAD		BFDD			HOT SAX			RAAD		BFDD			HOT SAX			RAAD		BFDD			HOT SAX			RAAD	
	40%	80%	Car.	40%	80%	Car.	80%	Car.	40%	80%	Car.	40%	80%	Car.	80%	Car.	40%	80%	Car.	40%	80%	Car.	80%	Car.	40%	80%	Car.	40%	80%	Car.	80%	Car.	40%	80%	Car.	40%	80%	Car.	80%	Car.
MITDB L = 554	*85.41 *	*85.41 *	*0 *	*91.17 *	*91.17 *	*0 *	***99.10***	***99.10***	***100***	***100***	*0 *	***100***	***100***	*0 *	***100***	***100***	***100***	***100***	*0 *	*50 *	*50 *	*0 *	***100***	***100***	***100***	***100***	*87.50 *	*87.50 *	*87.50 *	*75 *	***100***	***100***	***0***	***0***	*12.50 *	*12.50 *	*12.50 *	*25 *	***0***	***0***
ITDB *L* = 146																																								
Best	75.84	0	0	80.48	40.48	0	**89.11**	**89.11**	**100**	0	0	**100**	50	0	**100**	**100**	**100**	0	0	**100**	25	0	**100**	**100**	**100**	77.78	77.78	**100**	77.78	77.78	**100**	**100**	**0**	22.22	22.22	**0**	22.22	22.22	**0**	**0**
*μ*	74.51	0	0	71.53	20.24	0	**89.11**	**89.11**	**100**	0	0	**100**	25	0	**100**	**100**	83.34	0	0	75	12.50	0	**100**	**100**	94.45	72.23	72.23	88.89	72.23	66.67	**100**	**100**	5.56	27.78	27.78	11.11	27.78	33.33	**0**	**0**
SD	1.32	0	0	8.95	20.24	0	0	0	0	0	0	0	25	0	0	0	16.67	0	0	25	12.50	0	0	0	5.56	5.56	5.56	11.11	5.56	11.11	0	0	5.56	5.56	5.56	11.11	5.56	11.11	0	0
ITDB *L* = 154																																								
Best	73.19	0	0	87.88	87.88	0	**89.11**	**89.11**	**100**	0	0	**100**	**100**	0	**100**	**100**	**100**	0	0	**100**	**100**	0	**100**	**100**	**100**	77.78	77.78	**100**	**100**	77.78	**100**	**100**	**0**	22.22	22.22	**0**	**0**	22.22	**0**	**0**
*μ*	70	0	0	79.74	65.71	0	**89.11**	**89.11**	**100**	0	0	**100**	75	0	**100**	**100**	83.34	0	0	**100**	75	0	**100**	**100**	94.45	72.23	72.23	**100**	94.44	77.78	**100**	**100**	5.56	27.78	27.78	**0**	5.56	22.22	**0**	**0**
SD	3.19	0	0	8.14	2.17	0	0	0	0	0	0	0	25	0	0	0	16.67	0	0	0	25	0	0	0	5.56	5.56	5.56	0	5.56	0	0	0	5.56	5.56	5.56	0	5.56	0	0	0
INCART DB02 *L* = 217																																								
Best	*89.79 *	*89.79 *	*0 *	*79.67 *	*60.95 *	*0 *	***96.13***	***96.13***	***100***	***100***	*0 *	***100***	*66.67 *	*0 *	***100***	***100***	***100***	***100***	*0 *	*75 *	*25 *	*0 *	***100***	***100***	***100***	***100***	*72.73 *	*90.91 *	*72.73 *	*63.64 *	***100***	***100***	***0***	***0***	*27.27 *	*9.09 *	*27.27 *	*36.36 *	***0***	***0***
*μ*	*79.21 *	*60.90 *	*0 *	*64.44 *	*27.36 *	*0 *	***96.13***	***96.13***	*97.22 *	*69.44 *	*0 *	*91.67 *	*30.55 *	*0 *	***100***	***100***	*71.34 *	*52.51 *	*0 *	*47.84 *	*15.30 *	*0 *	***100***	***100***	*83.34 *	*75.76 *	*56.82 *	*69.70 *	*53.03 *	*44.70 *	***100***	***100***	*16.67 *	*24.24 *	*43.18 *	*30.30 *	*46.97 *	*55.31 *	***0***	***0***
SD	*10.19 *	*25.18 *	*0 *	*11.62 *	*15.01 *	*0 *	*0 *	*0 *	*9.21 *	*28.73 *	*0 *	*14.43 *	*16.43 *	*0 *	*0 *	*0 *	*26.92 *	*31.75 *	*0 *	*13.79 *	*7.75 *	*0 *	*0 *	*0 *	*17.34 *	*19.75 *	*17.06 *	*14.05 *	*12.77 *	*14.11 *	*0 *	*0 *	*17.34 *	*19.76 *	*17.06 *	*14.05 *	*12.77 *	*14.11 *	*0 *	*0 *
INCART DB02 *L* = 223																																								
Best	*89.79 *	*89.79 *	*0 *	*91.67 *	*91.67 *	*0 *	***96.13***	***96.13***	***100***	***100***	*0 *	***100***	***100***	*0 *	***100***	***100***	***100***	***100***	*0 *	*60 *	*50 *	*0 *	***100***	***100***	***100***	***100***	*72.73 *	*81.82 *	*72.73 *	*63.64 *	***100***	***100***	***0***	***0***	*27.27 *	*18.18 *	*27.27 *	*36.36 *	***0***	***0***
*μ*	*80.78 *	*60.69 *	*0 *	*59.45 *	*20.12 *	*0 *	***96.13***	***96.13***	***100***	*69.44 *	*0 *	*86.11 *	*22.22 *	*0 *	***100***	***100***	*72.80 *	*54.70 *	*0 *	*43.50 *	*11.46 *	*0 *	***100***	***100***	*84.85 *	*76.52 *	*57.58 *	*68.18 *	*50.76 *	*44.70 *	***100***	***100***	*15.15 *	*23.48 *	*42.42 *	*31.82 *	*49.25 *	*55.30 *	***0***	***0***
SD	*6.60 *	*25.31 *	*0 *	*15.97 *	*25.89 *	*0 *	*0 *	*0 *	*0 *	*28.73 *	*0 *	*16.43 *	*28.33 *	*0 *	*0 *	*0 *	*24.57 *	*35.29 *	*0 *	*9.49 *	*14.55 *	*0 *	*0 *	*0 *	*15 *	*20.48 *	*15 *	*10.16 *	*13.10 *	*10.14 *	*0 *	*0 *	*15 *	*20.48 *	*15 *	*10.17 *	*13.10 *	*10.14 *	*0 *	*0 *
INCART DB03 *L* = 212																																								
Best	83.11	83.11	0	76.55	40.64	23.56	**95.44**	**95.44**	**100**	**100**	0	**100**	50	25	**100**	**100**	**100**	75	0	**100**	22.22	14.29	**100**	**100**	**100**	90	60	**100**	60	60	**100**	**100**	**0**	10	40	**0**	40	40	**0**	**0**
*μ*	78.70	45.73	0	55.58	14.74	3.93	**95.44**	**95.44**	**100**	54.17	0	83.33	16.67	4.17	**100**	**100**	66.98	36.27	0	50.12	8.63	2.12	**100**	**100**	75	56.67	35	63.33	36.67	31.67	**100**	**100**	25	43.33	65	36.67	63.33	68.33	**0**	**0**
SD	3.79	18.52	0	13.11	13.36	8.78	0	0	0	22.44	0	15.59	15.59	9.32	0	0	20.95	17.76	0	17.07	7.76	4.78	0	0	17.08	18.86	17.08	14.91	11.79	14.04	0	0	17.08	18.86	17.08	14.91	11.79	14.04	0	0
INCART DB03 *L* = 223																																								
Best	83.44	83.44	0	70.43	66.42	25	**95.44**	**95.44**	**100**	**100**	0	**100**	75	25	**100**	**100**	**100**	60	0	80	37.50	12.50	**100**	**100**	**100**	80	60	90	60	50	**100**	**100**	**0**	20	40	10	40	50	**0**	**0**
*μ*	78.54	49.28	0	54.01	17.28	2.08	**95.44**	**95.44**	**100**	58.33	0	77.08	18.75	2.08	**100**	**100**	63.93	37.38	0	44.93	10.51	1.04	**100**	**100**	74.17	57.50	34.17	57.50	34.17	27.50	**100**	**100**	25.83	42.50	65.83	42.50	65.83	72.50	**0**	**0**
SD	4.31	17.33	0	9.42	18.60	6.91	0	0	0	21.24	0	16	20.73	6.91	0	0	15.43	15.02	0	17.95	11.08	3.45	0	0	13.82	16.39	13.82	17.85	14.98	12.99	0	0	13.82	16.39	13.82	17.85	14.98	12.99	0	0
INCART DB03 *L* = 234																																								
Best	93.22	93.22	0	86.56	69.61	0	**95.44**	**95.44**	**100**	**100**	0	**100**	75	0	**100**	**100**	**100**	60	0	57.14	37.50	0	**100**	**100**	**100**	80	60	70	50	40	**100**	**100**	**0**	20	40	30	50	60	**0**	**0**
*μ*	79.73	64.92	0	59.36	31.93	0	**95.44**	**95.44**	95.83	75	0	79.17	35.42	0	**100**	**100**	58.35	42.93	0	42.26	18.95	0	**100**	**100**	67.50	59.17	29.17	56.67	39.17	25	**100**	**100**	32.50	40.83	70.83	43.33	60.83	75	**0**	**0**
SD	8.27	19.44	0	17.60	17.56	0	0	0	9.32	22.82	0	19.98	18.98	0	0	0	18.51	9.79	0	10.55	9.73	0	0	0	18.31	13.20	18	8.50	9.54	6.45	0	0	18.31	13.20	18.01	8.50	9.54	6.45	0	0
INCART DB04 *L* = 203																																								
Best	*86.70 *	*86.70 *	*0 *	*69.46 *	*0 *	*0 *	***95.07***	***95.07***	***100***	***100***	*0 *	***100***	*0 *	*0 *	***100***	***100***	***100***	***100***	*0 *	***100***	*0 *	*0 *	***100***	***100***	***100***	***100***	*92.86 *	***100***	*92.86 *	*92.86 *	***100***	***100***	***0***	***0***	*7.14 *	***0***	*7.14 *	*7.14 *	***0***	***0***
*μ*	*72.70 *	*35.55 *	*0 *	*48.03 *	*0 *	*0 *	***95.07***	***95.07***	***100***	*41.67 *	*0 *	*83.33 *	*0 *	*0 *	***100***	***100***	*52.18 *	*28.06 *	*0 *	*56.94 *	*0 *	*0 *	***100***	***100***	*77.98 *	*73.81 *	*70.84 *	*91.67 *	*85.71 *	*85.71 *	***100***	***100***	*22.02 *	*26.19 *	*29.17 *	*8.33 *	*14.29 *	*14.29 *	***0***	***0***
SD	*11.42 *	*42.07 *	*0 *	*21.99 *	*0 *	*0 *	*0 *	*0 *	*0 *	*49.30 *	*0 *	*37.27 *	*0 *	*0 *	*0 *	*0 *	*40.78 *	*42.06 *	*0 *	*39.21 *	*0 *	*0 *	*0 *	*0 *	*21.52 *	*23.02 *	*21.52 *	*8.16 *	*7.72 *	*7.72 *	*0 *	*0 *	*21.52 *	*23.02 *	*21.52 *	*8.16 *	*7.72 *	*7.72 *	*0 *	*0 *
INCART DB05 *L* = 126																																								
Best	**100**	**100**	**0**	94.78	94.78	**0**	86.96	**0**	**100**	**100**	**0**	**100**	**100**	**0**	**100**	**0**	**100**	**100**	**0**	50	25	**0**	50	**0**	**100**	**100**	90	90	90	90	90	80	**0**	**0**	**10**	10	10	**10**	10	20
*μ*	64.34	41.90	**0**	43.08	16.52	**0**	**86.96**	**0**	81.81	45.45	0	63.64	18.18	**0**	**100**	**0**	20.84	14.77	**0**	21.82	4.09	0	**50**	**0**	50	46.36	41.81	70.91	66.36	64.55	**90**	**80**	50	53.64	58.18	29.09	33.64	35.45	**10**	**20**
SD	33.78	46.04	0	34.75	35.08	0	0	0	38.57	49.79	0	48.10	85.57	0	0	0	25.89	27.94	0	19.80	8.74	0	0	0	18.58	19.67	18	16.76	14.94	15.59	0	0	18.59	19.67	17.92	16.76	14.94	15.59	0	0

**Table 6:** The best value, mean (*μ*) and standard deviation (SD) of AoD, sensitivity, positive predictive value, specificity, and false alarm results obtained by RAAD and BitClusterDiscord with various overlap criteria. Bold figures denote the wining algorithms.

	AoD					Sensitivity					Positive predictive value					Specificity					False alarm rate				
	BitClusterDiscord			RAAD		BitClusterDiscord			RAAD		BitClusterDiscord			RAAD		BitClusterDiscord			RAAD		BitClusterDiscord			RAAD	
	40%	80%	Car.	80%	Car.	40%	80%	Car.	80%	Car.	40%	80%	Car.	80%	Car.	40%	80%	Car.	80%	Car.	40%	80%	Car.	80%	Car.
MITDB L = 554	*85.59 *	*85.59 *	*0 *	***99.10***	***99.10***	***100***	***100***	*0 *	***100***	***100***	***100***	***100***	*0 *	***100***	***100***	***100***	***100***	*87.5 *	***100***	***100***	***0***	***0***	*12.5 *	***0***	***0***
ITDB *L* = 146																									
Best	62.79	0	0	**89.11**	**89.11**	**100**	0	0	**100**	**100**	**100**	0	0	**100**	**100**	**100**	77.78	77.78	**100**	**100**	**0**	22.22	22.22	**0**	**0**
*μ*	58.56	0	0	**89.11**	**89.11**	**100**	0	0	**100**	**100**	**100**	0	0	**100**	**100**	**100**	77.78	77.78	**100**	**100**	**0**	22.22	22.22	**0**	**0**
SD	4.23	0	0	0	0	0	0	0	0	0	0	0	0	0	0	0	0	0	0	0	0	0	0	0	0
ITDB *L* = 154																									
Best	83.76	83.76	0	**89.11**	**89.11**	**100**	**100**	0	**100**	**100**	**100**	**100**	0	**100**	**100**	**100**	**100**	77.78	**100**	**100**	**0**	22.22	22.22	**0**	**0**
*μ*	69.7	41.88	0	**89.11**	**89.11**	**100**	50	0	**100**	**100**	**100**	50	0	**100**	**100**	**100**	88.89	77.78	**100**	**100**	**0**	11.11	22.22	**0**	**0**
SD	14.05	41.88	0	0	0	0	50	0	0	0	0	50	0	0	0	0	11.11	0	0	0	0	11.11	0	0	0
INCART DB02 *L* = 217																									
Best	*84.97 *	*61.05 *	*31.87 *	***96.13***	***96.13***	***100***	*66.67 *	*33.33 *	***100***	***100***	*75 *	*25 *	*12.5 *	***100***	***100***	*90.91 *	*63.64 *	*63.64 *	***100***	***100***	*45.46 *	*72.73 *	*72.73 *	***0***	***0***
*μ*	*48.98 *	*9.84 *	*2.66 *	***96.13***	***96.13***	*75 *	*11.11 *	*2.78 *	***100***	***100***	*41.76 *	*4.79 *	*1.04 *	***100***	***100***	*70.45 *	*53.03 *	*50.76 *	***100***	***100***	*29.55 *	*46.97 *	*49.24 *	***0***	***0***
SD	*23.74 *	*18.69 *	*8.81 *	*0 *	*0 *	*30.81 *	*20.79 *	*9.21 *	*0 *	*0 *	*18.62 *	*8.69 *	*3.45 *	*0 *	*0 *	*12.92 *	*12.77 *	*14.11 *	*0 *	*0 *	*12.92 *	*12.77 *	*14.11 *	*0 *	*0 *
INCART DB02 *L* = 223																									
Best	*85.82 *	*61.45 *	*0 *	***96.13***	***96.13***	***100***	*66.67 *	*0 *	***100***	***100***	*75 *	*33.33 *	*0 *	***100***	***100***	*90.91 *	*81.82 *	*72.73 *	***100***	***100***	*54.55 *	*81.82 *	*81.82 *	***0***	***0***
*μ*	*47.31 *	*9.79 *	*0 *	***96.13***	***96.13***	*72.22 *	*11.11 *	*0 *	***100***	***100***	*39.73 *	*6.2 *	*0 *	***100***	***100***	*68.94 *	*52.27 *	*49.24 *	***100***	***100***	*31.06 *	*47.73 *	*50.76 *	***0***	***0***
SD	*25.57 *	*18.7 *	*0 *	*0 *	*0 *	*35.57 *	*20.79 *	*0 *	*0 *	*0 *	*22.05 *	*11.63 *	*0 *	*0 *	*0 *	*14.11 *	*16.65 *	*16.79 *	*0 *	*0 *	*14.11 *	*16.65 *	*16.79 *	*0 *	*0 *
INCART DB03 *L* = 212																									
Best	74.85	45.54	0	**95.44**	**95.44**	**100**	50	0	**100**	**100**	66.67	25	0	**100**	**100**	80	40	40	**100**	**100**	50	90	90	**0**	**0**
*μ*	62.2	9.4	0	**95.44**	**95.44**	93.75	10.42	0	**100**	**100**	52.51	5.36	0	**100**	**100**	65	31.67	27.5	**100**	**100**	35	68.33	72.5	**0**	**0**
SD	9.2	14.5	0	0	0	10.83	16	0	0	0	8.61	8.15	0	0	0	9.57	9.86	10.1	0	0	9.57	9.86	10.1	0	0
INCART DB03 *L* = 223																									
Best	76.34	45.31	0	**95.44**	**95.44**	**100**	50	0	**100**	**100**	66.67	25	0	**100**	**100**	80	50	50	**100**	**100**	60	90	90	**0**	**0**
*μ*	65.67	18.06	0	**95.44**	**95.44**	93.75	20.83	0	**100**	**100**	52.55	10.91	0	**100**	**100**	64.17	35	26.67	**100**	**100**	35.83	65	73.33	**0**	**0**
SD	10.32	17.37	0	0	0	10.83	19.98	0	0	0	10.06	10.17	0	0	0	12.56	12.58	12.47	0	0	12.56	12.58	12.47	0	0
INCART DB03 *L* = 234																									
Best	79.57	63.48	0	**95.44**	**95.44**	**100**	75	0	**100**	**100**	66.67	42.86	0	**100**	**100**	80	60	40	**100**	**100**	50	80	80	**0**	**0**
*μ*	67.62	38.61	0	**95.44**	**95.44**	91.67	45.83	0	**100**	**100**	51.34	25.35	0	**100**	**100**	64.17	45.83	27.5	**100**	**100**	35.83	54.17	72.5	**0**	**0**
SD	8.16	19	0	0	0	11.79	22.44	0	0	0	9.67	11.71	0	0	0	9.54	11.15	8.29	0	0	9.54	11.15	8.29	0	0
INCART DB04 *L* = 203																									
Best	*95 *	*95 *	*0 *	***95.07***	***95.07***	***100***	***100***	*0 *	***100***	***100***	***100***	*33.33 *	*0 *	***100***	***100***	***100***	*92.86 *	*92.86 *	***100***	***100***	*21.43 *	*28.57 *	*28.57 *	***0***	***0***
*μ*	*57.64 *	*15.56 *	*0 *	***95.07***	***95.07***	*91.67 *	*16.67 *	*0 *	***100***	***100***	*73.61 *	*4.86 *	*0 *	***100***	***100***	*94.05 *	*88.69 *	*87.5 *	***100***	***100***	*5.95 *	*11.31 *	*12.5 *	***0***	***0***
SD	*23.22 *	*34.8 *	*0 *	*0 *	*0 *	*27.64 *	*37.27 *	*0 *	*0 *	*0 *	*38.01 *	*11 *	*0 *	*0 *	*0 *	*8.67 *	*6.81 *	*8.31 *	*0 *	*0 *	*8.67 *	*6.81 *	*8.31 *	*0 *	*0 *
INCART DB05 *L* = 126																									
Best	94.78	94.78	0	86.96	**0**	**100**	**100**	0	**100**	**0**	50	16.67	0	50	**0**	90	80	80	90	80	80	80	80	10	20
*μ*	54.15	16.44	0	**86.96**	**0**	81.82	18.18	0	**100**	**0**	19.71	3.03	0	**50**	**0**	51.82	45.45	43.64	**90**	80	48.18	54.55	56.36	**10**	**20**
SD	28.97	34.93	0	0	0	38.57	38.57	0	0	0	15.73	6.43	0	0	0	21.67	19.71	19.67	0	0	21.67	19.71	19.67	0	0
